# Health-related quality of life across disease stages in patients with amyotrophic lateral sclerosis: results from a real-world survey

**DOI:** 10.1007/s00415-023-12141-y

**Published:** 2024-01-11

**Authors:** Katie Stenson, T. E. Fecteau, L. O’Callaghan, P. Bryden, J. Mellor, J. Wright, L. Earl, O. Thomas, H. Iqbal, S. Barlow, S. Parvanta

**Affiliations:** 1 Biogen, Cambridge, MA USA; 2 Adelphi Real World, Bollington, UK; 3 The ALS Association, Arlington, VA USA; 4 Present Address: Sage Therapeutics, Boston, MA USA

**Keywords:** Amyotrophic lateral sclerosis, Quality of life, Patient-reported outcomes, Disease progression, Real-world evidence

## Abstract

**Background:**

Amyotrophic lateral sclerosis (ALS) is characterized by a rapid disease course, with disease severity being associated with declining health-related quality of life (HRQoL) in persons living with ALS (pALS). The main objective of this study was to assess the impact of disease progression on HRQoL across King’s, Milano-Torino Staging (MiToS), and physician-judgement clinical staging. Additionally, we evaluated the impact of the disease on the HRQoL of care partners (cALS).

**Methods:**

Data were sourced from the Adelphi ALS Disease Specific Programme (DSP)™, a cross-sectional survey of neurologists, pALS and cALS presenting in a real-world clinical setting between July 2020 and March 2021 in Europe and the United States.

**Results:**

Neurologists (*n* = 142) provided data for 880 pALS. There were significant negative correlations between all three clinical staging systems and EuroQol (European Quality of Life) Five Dimension Five Level Scale (EQ-5D-5L) utility scores and visual analogue scale (VAS) ratings. Although not all differences were significant, 5-item Amyotrophic Lateral Sclerosis Assessment Questionnaire (ALSAQ-5) scores showed a stepwise increase in HRQoL impairment at each stage of the disease regardless of the staging system. At later stages, high levels of fatigue and substantial activity impairment were reported. As pALS disease states progressed, cALS also experienced a decline in HRQoL and increased burden.

**Conclusions:**

Across outcomes, pALS and cALS generally reported worse outcomes at later stages of the disease, highlighting an unmet need in this population for strategies to maximise QoL despite disease progression. Recognition and treatment of symptoms such as pain and fatigue may lead to improved outcomes for pALS and cALS.

**Supplementary Information:**

The online version contains supplementary material available at 10.1007/s00415-023-12141-y.

## Background

Amyotrophic lateral sclerosis (ALS) is a rare, heterogenous, neurodegenerative disease, characterized by progressive loss of muscle function, and ultimately death [1]. ALS is thought to be caused by a combination of genetic and environmental factors [2, 3]. ALS has a median survival of around 3 years after symptom onset, with respiratory failure being the cause of death in most cases [1].

The King’s staging and Milano-Torino Staging (MiToS) systems are established clinical staging systems used to monitor ALS disease progression [4]. The King’s system assesses any loss of independence in affected regions (bulbar, lower limb, upper limb) and requirement for assistive devices (gastrostomy and tracheostomy), whereas MiToS assesses complete loss of independence in four key domains (bulbar, gross motor, fine motor, and respiratory function) [4, 5]. The King’s system typically differentiates well in early-stage disease, with MiToS being better in differentiating mid- to late-stages [4, 6]. Both staging criteria are derived or can be mapped from Revised Amyotrophic Lateral Sclerosis Functional Rating (ALSFRS-R) scores [7, 8]. Additionally, persons living with ALS (pALS) can be staged as ‘early’/’mid’/’late’ by leveraging a physician’s treatment experience to outline the current stage of the disease in relation to the expected disease duration for that patient. This physician-judgment staging method is more subjective but is useful when ALSFRS-R scores are not available.

Physical impairment and progression rate of physical deterioration in ALS have a significant impact on emotional well-being and there is a correlation between disease severity and/or decreasing physical function and declining health-related quality of life (HRQoL) [9, 10]. A recent systematic literature review investigating the burden of ALS confirmed that pALS experienced poor QoL and loss of function, which deteriorated with disease progression [11]. The burden of symptoms was high, with patients experiencing various debilitating symptoms, with fatigue, depression, and pain being the most frequently reported [11]. On the other hand, there are reports in the literature of relatively well-maintained QoL despite physical function decline [12–14]. Interestingly, Vázquez Medrano and colleagues [15] found that disease progression but not physical state per se determined mental well-being in ALS.

Depending on the severity of the disease, care partners of pALS (cALS) who are not providing care under a professional contract may spend most of the day providing care, resulting in substantial humanistic and economic burden, with associated depression and reduced QoL [11, 16]. A cross-sectional survey in Germany revealed that costs of informal care (i.e., care provided by non-professional care partners, mainly family members) represented nearly half of all costs of illness in ALS [17]. pALS and cALS are also less likely to be gainfully employed [18], and this productivity loss may have a significant financial impact on pALS and cALS [18].

While outcome measures such as ALSFRS-R capture changes in physical functioning, tools such as the EuroQol (European Quality of Life) Five Dimension Five Level Scale (EQ-5D-5L) [19] or disease-specific patient-reported outcomes (PRO) measures such as the 40-item Amyotrophic Lateral Sclerosis Assessment Questionnaire (ALSAQ-40) and 5-item Amyotrophic Lateral Sclerosis Assessment Questionnaire (ALSAQ-5), can be utilized to assess well-being and quality of life as perceived by pALS and/or cALS.

Although longitudinal studies of QoL in pALS and cALS, using different instruments and varying in focus and conclusions, have been previously conducted [20–24], publications reporting real-world changes in QoL and other disease-specific PRO measures across the ALS disease course remain rare. Hence, the main objective of the present study was to assess the impact of disease progression on HRQoL across King’s, MiToS, and physician-judgement stages. Additionally, we evaluated the care partner burden and the impact of the disease on the HRQoL of cALS.

## Methods

Data were sourced from the Adelphi ALS Disease Specific Programme (DS)™, a large, prospective, cross-sectional survey of neurologists, pALS and cALS presenting in a real-world clinical setting. The survey was conducted between July 2020 and March 2021 in France, Germany, Italy, Spain, the United Kingdom (UK), and the United States (US). The survey methodology has been previously published and validated [25–27].

### Participants

Physicians were identified by local fieldwork agents using physician panels and publicly available lists and invited to participate if they had a primary specialty of neurology (general neurologist, neuromuscular specialist, or ALS specialist), were actively involved in the management of ALS, were seeing two or more pALS per month, and agreed to adhere to all survey rules and regulations.

pALS were aged ≥ 18 years at data collection and had a physician-confirmed diagnosis of ALS. cALS were aged ≥ 18 years at data collection and were self-identified as a care partner.

Neurologists completed detailed electronic patient record forms (PRFs) for the next 1–10 consecutive consulting pALS; the PRFs included pALS demographics and clinical characteristics, including ALSFRS-R data, which were used to derive King’s and MiToS staging. Neurologists also classified pALS as either ‘early’, ‘middle’, or ‘late’ stage ALS, per their clinical judgement. These pALS were invited to voluntarily complete a ‘pen and paper’ patient self-completion (PSC) form assessing HRQoL, including the ALSAQ-5, EQ-5D-5L, Fatigue Severity Scale (FSS), and Work Productivity and Activity Impairment Questionnaire (WPAI). cALS were able to help pALS complete the written form if needed.

If present with pALS at the point of consultation, cALS were invited to voluntarily complete a ‘pen and paper’ care partner self-completion (CSC) form, which included the Zarit Burden Interview (ZBI), EQ-5D-5L (cALS perspective on pALS’ general health), EQ-5D-5L (cALS perspective on their own general health), and WPAI.

### Assessment tools

The EQ‐5D‐5L is a 5-dimension questionnaire used to assess the decline in health status in various conditions [19, 28, 29], including ALS [30]. It is widely used to determine the impact of the disease on the HRQoL of patients and care partners in five different domains: mobility, self-care, usual activities, pain/discomfort, and anxiety/depression. It is useful for calculating quality-adjusted life-years and facilitating comparisons of health technologies between different diseases [30]. While not disease-specific, the EQ-5D-5L covers aspects that are relevant to ALS, with domains that are similar to those used in disease-specific assessments such as the ALSAQ-5 (e.g., mobility in the EQ-5D-5L versus physical mobility in the ALSAQ-5, usual activities versus daily activities, anxiety/depression versus emotional well-being).

For each domain, the response options (levels) on the EQ-5D-5L are ‘no’, ‘slight’, ‘moderate’, ‘severe’, and ‘extreme problems/unable to’, and health states are converted into a single index ‘utility’ score using a scoring algorithm/value set/preference weight based on public preferences [31]. In line with guidance from the National Institute for Health and Care Excellence [32], the current study calculated utility values by mapping the 5L descriptive system data onto the 3L using the Hernández-Alava UK value set [32–34].

In addition to the EQ-5D-5L questionnaire, a vertical visual analogue scale (VAS) is used to record the patient’s self-rated health (and/or care partner’s rating of patient’s health, as well as their own self-rated health), from 0 (worst imaginable health state) to 100 (best imaginable health state) [19].

The 40-item Amyotrophic Lateral Sclerosis Assessment Questionnaire (ALSAQ-40) is a 40-item questionnaire used to specifically measure the HRQoL of pALS and motor neuron disease [35]. The 5-item ALSAQ (ALSAQ-5) used in the present study is an abbreviated version of the ALSAQ-40, with five items (physical mobility, activities of daily living, eating/drinking, communication, and emotional functioning), each representing one domain of the longer form [35]. The ALSAQ-5 is useful in surveys and trials, as it produces very similar results to the ALSAQ-40 [35]. The overall ALSAQ-5 score ranges from 0 (best imaginable health state) to 100 (worst imaginable health state).

The FSS is a 9-item patient-reported questionnaire assessing fatigue across three domains: life participation, sleep, and daily activities [36]. While the FSS was not developed specifically for ALS, these domains are relevant to pALS. Each question is scored from 1 (completely disagree) to 7 (completely agree), for a total possible score of 63. Higher scores indicate greater fatigue in everyday life and a total score of > 40 suggests clinically significant fatigue [37].

The WPAI is a 6-item questionnaire used to assess impairment in work-related productivity and daily activities due to health [38, 39]. It measures employment and rates of absenteeism, work productivity loss, and impairment in regular daily activities within the past 7 days. The WPAI is a non-disease-specific questionnaire, but it covers areas of relevance to pALS. WPAI outcomes are expressed as impairment percentages from 0 to 100, with higher percentages indicating greater impairment and lower productivity. In this study, WPAI work impairment variables were excluded due to the small sample of patients in employment, and therefore the WPAI results focus on activity impairment.

The ZBI is a 12-item self-administered questionnaire used to measure care partner burden by evaluating disease impact on care partners [40, 41]. The ZBI assesses the care partner overall QoL, emotional well-being, and impact on social and family relationships. Each item is rated on a scale of 0–4, for a maximum possible score of 48. A total score greater than 17 indicates a high burden.

### Statistical analysis

All data were aggregated, de-identified and anonymized before receipt by Adelphi Real World. To link physician-reported data for disease stage with pALS/cALS-reported outcomes, results were derived from matched PRF-PSC, or PRF-CSC pairs.

Data were analyzed for the countries combined (i.e., aggregated overall data) and are interpreted at a global level. Descriptive statistics were used for demographics and clinical characteristics. Correlations of outcomes with King’s and MiToS stages were assessed through linear regression and were adjusted for age, sex, body mass index (BMI), and number of comorbidities. Adjusted marginal means were reported. Pairwise comparisons between disease stages were conducted using Wald tests. For ALSAQ-5, FSS and WPAI, MiToS stage 4 was excluded, due to small group sizes (*n* = 1 or *n* = 2), which did not allow for regression modelling.

Bivariate comparisons across disease stages were conducted using chi-squared and Kruskal–Wallis tests. Pairwise analysis between groups was conducted using Fisher’s exact and Mann–Whitney tests. Comparisons of pALS-reported versus cALS-reported (i.e., proxy) EQ-5D-5L and VAS outcomes were conducted using paired *t*-tests.

Any pALS with missing data for a particular variable was removed from all analyses involving that variable, but pALS who were removed from one set of analysis were still eligible for inclusion in other analyses. It should also be noted that because the MiToS system has a stage 0 (which captures pALS with functional involvement but not yet full loss of any given function), all pALS were included in the analysis regardless of their ALSFRS-R scores. In contrast, the King’s system does not have a stage 0 equivalent and hence pALS who did not meet any of the staging criteria were excluded from the analysis.

Analyses were performed using Stata 17.0 [42].

## Results

### Demographics and characteristics

A total of 142 neurologists completed PRFs for 880 pALS. Of these, 172 pALS had self-reported and/or cALS-reported (by proxy) data. pALS demographics and characteristics are summarized in Table 1. Overall, the mean (standard deviation [SD]) age was 60.8 (11.5) years, 60.5% (*n* = 104) were male, and 93.6% (*n* = 161) were White/Caucasian. The mean (SD) time since ALS diagnosis was 22.0 (29.2) months. cALS demographics are summarized in Table 2. Partners/spouses made up 72.8% of cALS (*n* = 59).

**Table 1:** pALS demographics and characteristics

	*n* = 172
Country, *n* (%)	
France	33 (19.2)
Germany	19 (11.1)
Italy	27 (15.7)
Spain	42 (24.4)
UK	8 (4.7)
US	43 (25.0)
Age (years)	
Mean (SD)	60.8 (11.5)
Range	22.0, 90.0
Gender (male), *n* (%)	104 (60.5)
BMI	
Mean (SD)	23.7 (3.0)
Range	11.3, 29.4
Underweight, *n* (%)	9 (5.2)
Healthy weight, *n* (%)	106 (61.6)
Overweight, *n* (%)	57 (33.1)
Obese, *n* (%)	0 (0.0)
Ethnicity^a^, *n* (%)	
White/Caucasian	161 (93.6)
Hispanic/Latino/Latina	8 (4.7)
Other	2 (1.2)
Living circumstances^a^, *n* (%)	
Lives alone in own home	15 (8.8)
Lives with partner/spouse/immediate family	141 (82.5)
Lives with other family/friends	8 (4.7)
Lives in hospice	1 (0.6)
Lives in nursing home	5 (2.9)
Lives in assisted living residence/residential home	1 (0.6)
Employment status^b^, *n* (%)	
Working full time	13 (7.7)
Working part time	21 (12.4)
On long term sick leave	32 (18.8)
Homemaker	13 (7.7)
Student	1 (0.6)
Retired	81 (47.7)
Unemployed	9 (5.3)
Number of comorbidities	
Mean (SD)	1.5 (1.4)
Range	0.0, 7.0
Time since diagnosis (months)^c^	
Mean (SD)	22.0 (29.2)
Range	0.0, 221.4
ALSFRS-R score (at time of survey)	
Mean (SD)	32.7 (12.4)
Range	0.0, 48.0
ALS stage—physician judgement, *n* (%)	
Early stage	64 (37.2)
Middle stage	76 (44.2)
Late stage	32 (18.6)
ALS stage—MiToS, *n* (%)	
Stage 0	122 (70.9)
Stage 1	18 (10.5)
Stage 2	10 (5.8)
Stage 3	8 (4.7)
Stage 4	14 (8.1)
ALS stage—King’s^d^, *n* (%)	
Stage 1	29 (17.2)
Stage 2	27 (16.0)
Stage 3	61 (36.1)
Stage 4a/b	52 (30.8)

ALS, amyotrophic lateral sclerosis; ALSFRS-R, Revised Amyotrophic Lateral Sclerosis Functional Rating; BMI, body mass index; MiToS, Milano-Torino Staging; pALS, persons living with ALS; SD, standard deviation; UK, United Kingdom; US, United States

^a^*n* = 171 (1 patient missing data: “don’t know” response)

^b^*n* = 170 (2 patients missing data: “don’t know” response)

^c^*n* = 169 (3 patients missing data: “don’t know” response)

^d^*n* = 169 (3 patients unable to be assigned stage per the King’s algorithm—disease not advanced enough to meet any of the individual criteria)

**Table 2:** cALS demographics

	*n* = 82
Country, *n* (%)	
France	24 (29.3)
Germany	6 (7.3)
Italy	12 (14.6)
Spain	16 (19.5)
UK	3 (3.7)
US	21 (25.6)
Age (years)^a^	
Mean (SD)	58.8 (12.7)
Range	24.0, 85.0
Gender (female), *n* (%)	53 (64.6)
Relationship with patient^b^, *n* (%)	
Partner/spouse	59 (72.8)
Son/daughter	7 (8.6)
Sibling	6 (7.4)
Other family member	0 (0.0)
Friend/neighbour	2 (2.5)
Professional/paid care partner	7 (8.6)

cALS, care partners of persons living with ALS; SD, standard deviation; UK, United Kingdom; US, United States

^a^*n* = 80 (2 care partners missing data: did not respond to question)

^b^*n* = 81 (1 care partner missing data: did not respond to question)

Examining the proportion of pALS assigned to each of the King’s stages versus physician judgement, while there was variability, “early” stage pALS were most frequently placed at stages 1–2, “middle” at stage 3, and “late” at stage 4. In contrast, when examining MiToS versus physician judgement, it appeared that MiToS provided more granularity at later stages (Supplementary Table S1).

### Patient burden

#### EQ-5D-5L utility score

**Fig. 1:**
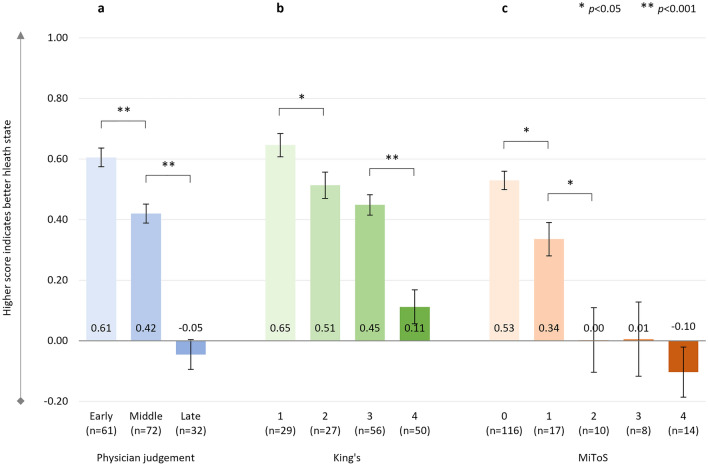
EQ-5D-5L utility score

There were significant negative correlations between physician-judged staging and EQ-5D-5L utility score (*r*^2^ = 0.480, *p* < 0.001, *n* = 165), with a significant decline in health status from ‘early’ to ‘middle’, and from ‘middle’ to ‘late’ disease stages (Fig. 1a). Significant negative correlations between King’s and MiToS disease stages and EQ-5D-5L utility scores were also observed: King’s staging and EQ-5D-5L utility score: *r*^2^ = 0.371, *p* < 0.001 (*n* = 162) (Fig. 1b); MiToS staging and EQ-5D-5L utility score: *r*^2^ = 0.461, *p* < 0.001 (*n* = 165) (Fig. 1c).

An additional analysis was conducted to compare pALS-reported versus cALS-reported (i.e., proxy) EQ-5D-5L utility scores for the 42 pALS who had both their own and a cALS perspective on the patient’s general health—this analysis showed similar utility scores in both groups (Supplementary Table S2). Domain-level results are shown in Supplementary Table S3. These indicated significant gradual worsening over the different stages regardless of the staging system for the individual domains of mobility, self-care, usual activities, and pain/discomfort. Results for anxiety/depression were less clear, however, indicated a significant gradual worsening over King’s staging.

#### EQ-5D-VAS

**Fig. 2:**
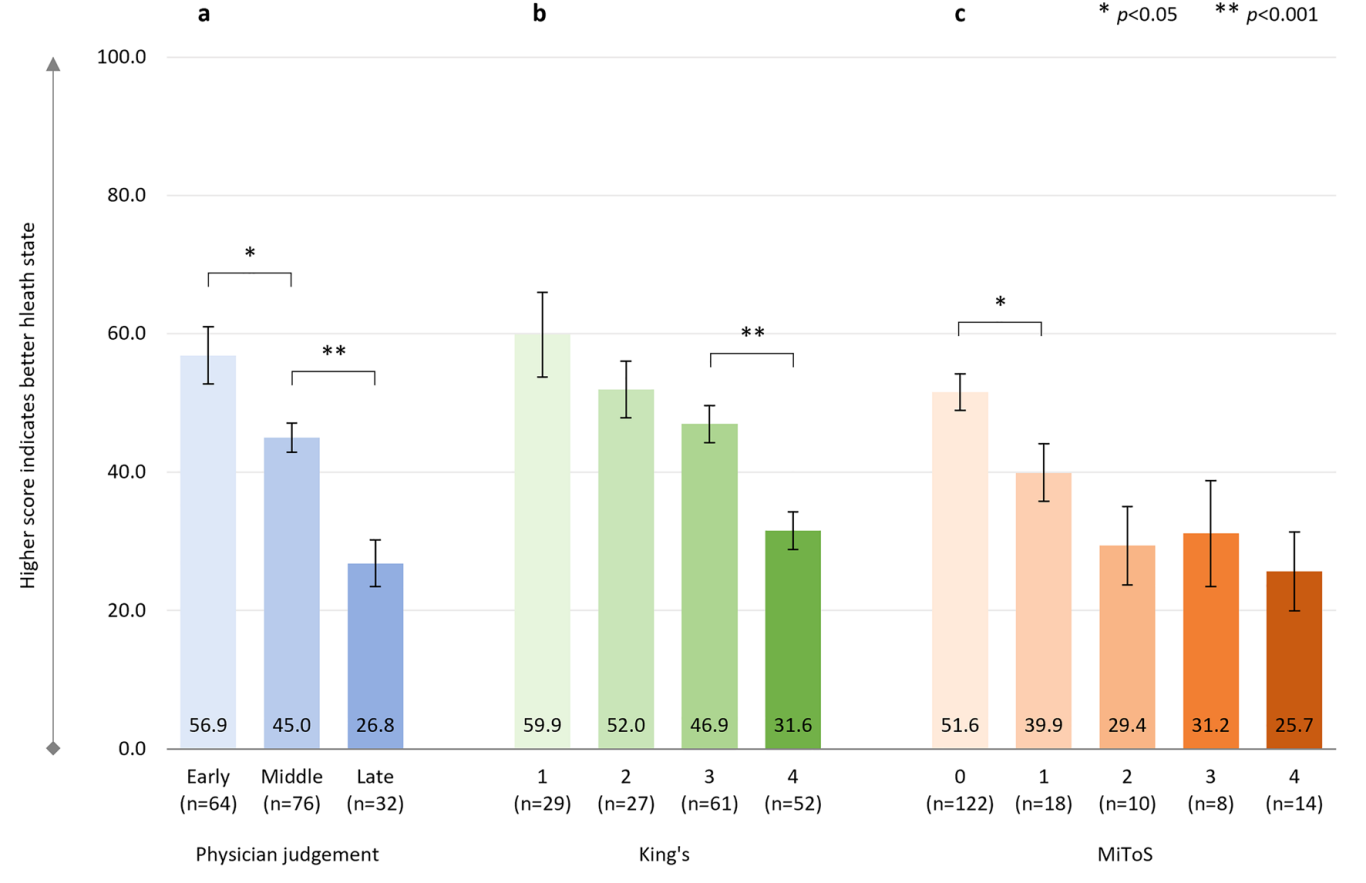
EQ-5D-VAS score

In addition, there were significant negative correlations between physician-judged staging and EQ-VAS score (*r*^2^ = 0.245, *p* < 0.001, *n* = 172), with a significant decline in health status from ‘early’ to ‘middle’, and from ‘middle’ to ‘late’ disease stages (Fig. 2a), and between King’s and MiToS disease stages and EQ-VAS scores: King’s staging and EQ-VAS score: *r*^2^ = 0.226, *p* < 0.001 (*n* = 169) (Fig. 2b); MiToS staging and EQ-VAS score: *r*^2^ = 0.201, *p* < 0.001 (*n* = 172) (Fig. 2c).

The additional analysis comparing pALS-reported versus cALS-reported (i.e., proxy) EQ-5D-VAS scores for the 42 pALS who had both their own and a cALS perspective on the patient’s general health showed similar VAS scores in both groups (Supplementary Table S3).

#### ALSAQ-5

**Fig. 3:**
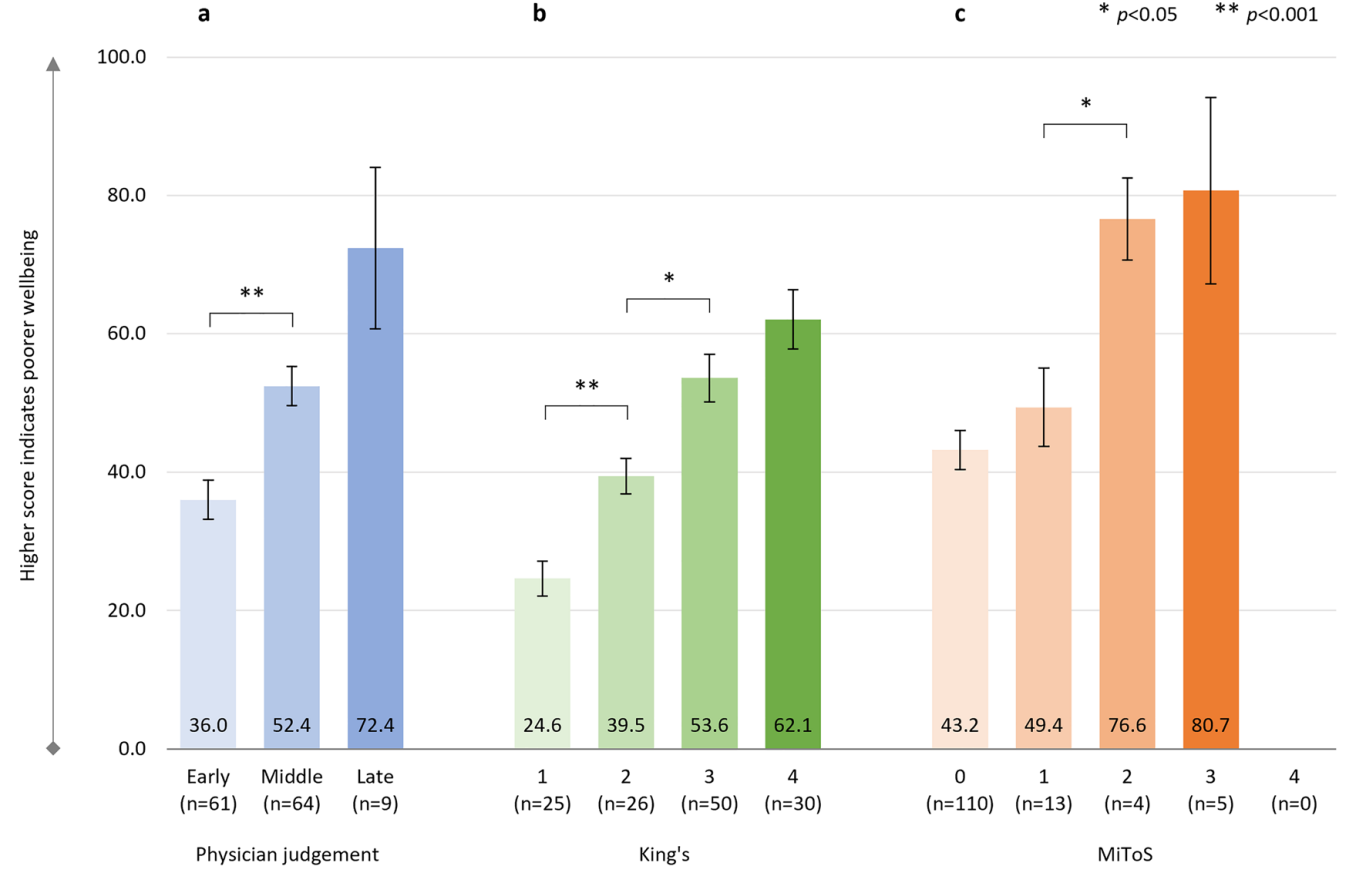
ALSAQ-5 score

There was a significant positive correlation between physician-judged staging and ALSAQ-5 score: *r*^2^ = 0.258, *p* < 0.001 (*n* = 134), with significantly greater impairment in HRQoL from ‘early’ to ‘middle’ disease stages (Fig. 3a). ALSAQ-5 scores were also significantly positively correlated with both King’s and MiToS stages: King’s staging and ALSAQ-5 score: *r*^2^ = 0.371, *p* < 0.001 (*n* = 131) (Fig. 3b); MiToS staging and ALSAQ-5 score: *r*^2^ = 0.193, *p* < 0.001 (*n* = 132) (Fig. 3c). ALSAQ-5 scores showed a stepwise increase in HRQoL impairment at each progressing stage for both King’s and MiToS staging systems, although not all these differences were significant.

Item-level results are shown in Supplementary Table S4 and generally, there was a significant consistent worsening of individual items (physical mobility, activities of daily living, eating/drinking, communication, and emotional functioning) over the different stages of all staging systems. An exception was emotional functioning across physician judgement stages, where no significant worsening was observed.

#### FSS

**Fig. 4:**
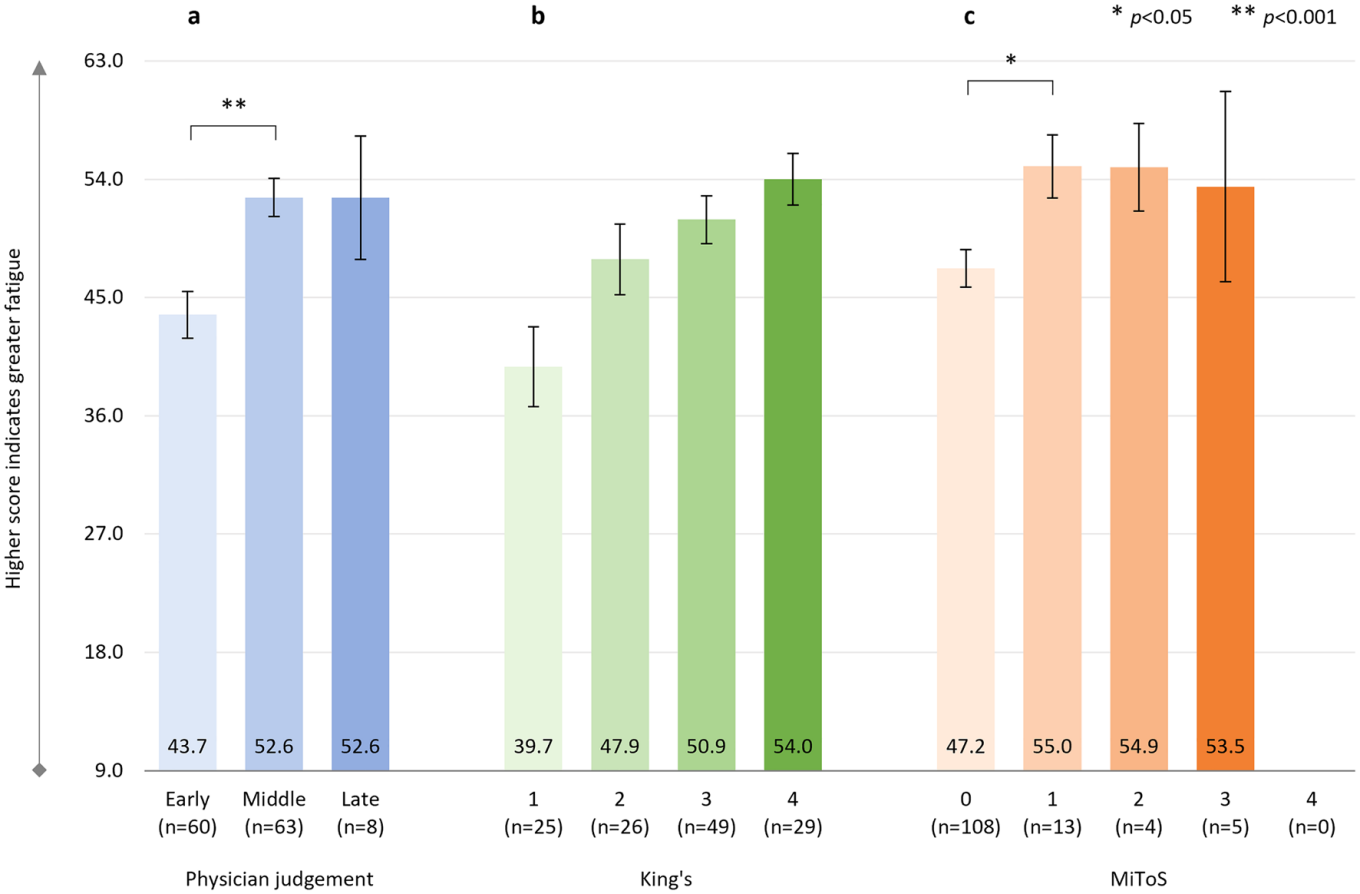
FSS score

A significant positive correlation between physician-judged staging and FSS score was observed: *r*^2^ = 0.225, *p* < 0.001 (*n* = 131) (Fig. 4a). Similarly, there was a significant positive correlation between King’s staging and FSS score: *r*^2^ = 0.234, *p* < 0.001 (*n* = 129) (Fig. 4b), and a significant non-linear correlation between MiToS staging and FSS score: *r*^2^ = 0.136, *p* = 0.011 (*n* = 130) (Fig. 4c). The mean levels of fatigue reported were all clinically significant (total FSS score > 40).

Item-level results are shown in Supplementary Table S5, with fairly consistent and in most cases significant worsening of all individual items of the FSS over different stages for all staging systems.

#### WPAI (activity impairment score)

**Fig. 5:**
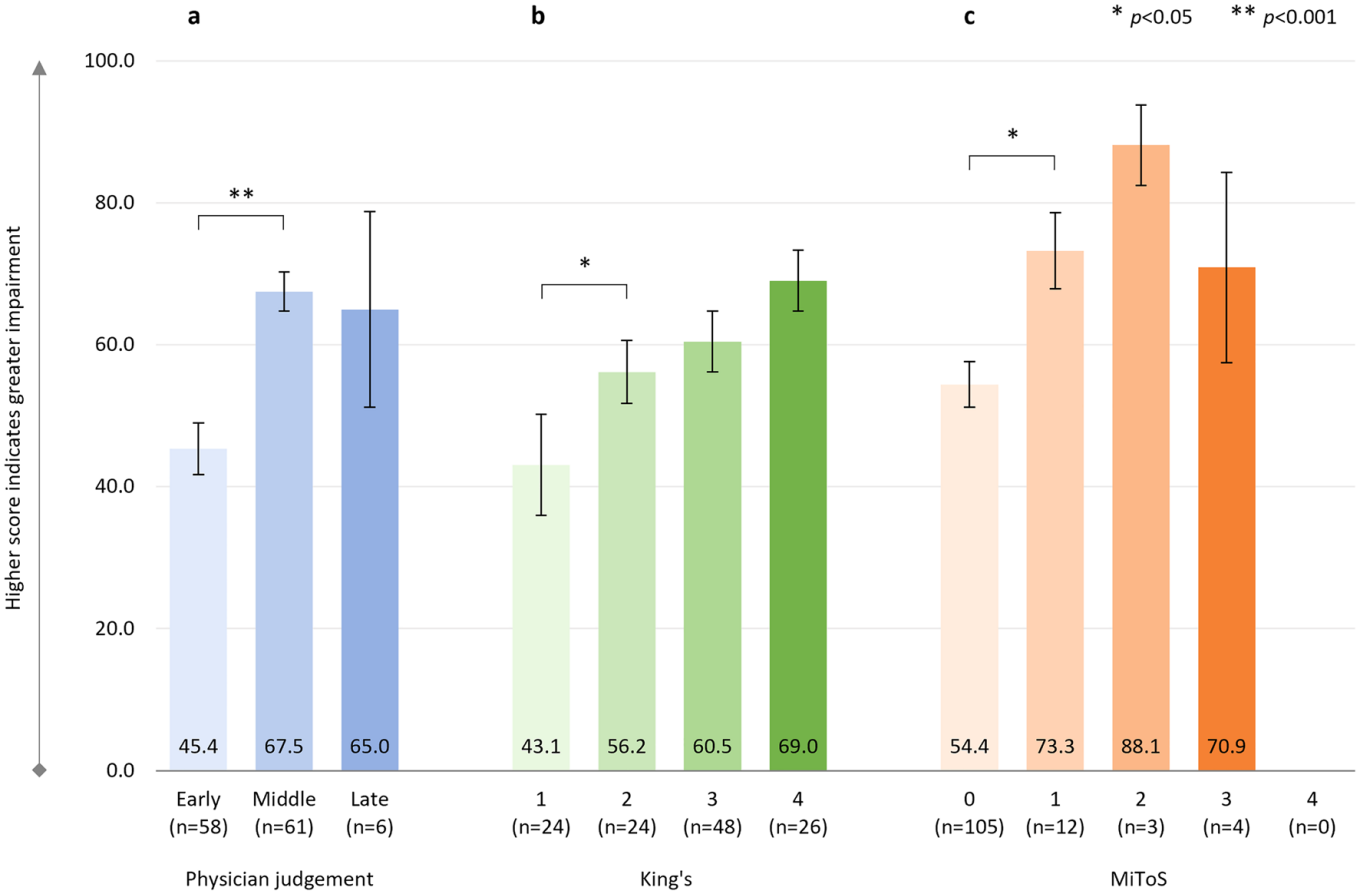
WPAI activity impairment score

A significant non-linear correlation between physician-judged staging and WPAI activity impairment score was observed: *r*^2^ = 0.270, *p* < 0.001 (*n* = 125), with significantly greater activity impairment from the ‘early’ to ‘middle’ disease stages (Fig. 5a). A significant non-linear correlation between MiToS disease staging and WPAI activity impairment score was also observed: *r*^2^ = 0.179, *p* < 0.001 (*n* = 124), with significantly greater activity impairment from stage 0 to stage 1 (Fig. 5b). Lastly, there was a significant positive correlation between King’s disease staging and WPAI activity impairment score: *r*^2^ = 0.184, *p* = 0.030 (*n* = 122), with significantly greater activity impairment from stage 1 to stage 2 (Fig. 5c).

### Care partner burden

#### ZBI

**Fig. 6:**
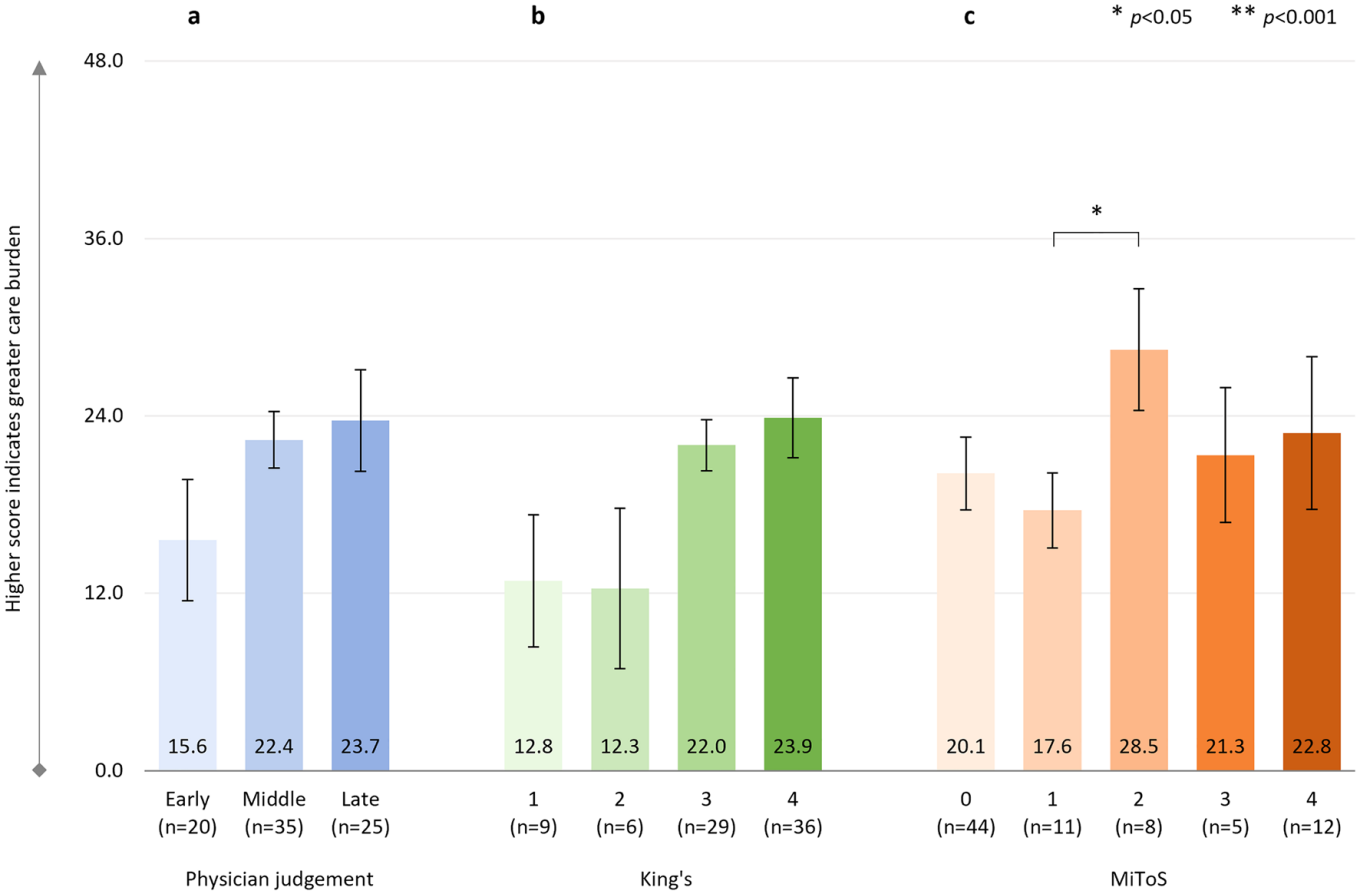
ZBI-12 score

There were no significant correlations between cALS ZBI-12 scores and any of the disease staging tools (Fig. 6a–c): physician judgement (*r*^2^ = 0.091, *p* = 0.178 [*n* = 80]), King’s staging (*r*^2^ = 0.141, *p* = 0.070 [*n* = 80]), or MiToS staging (*r*^2^ = 0.078, *p* = 0.178 [*n* = 80]).

Item-level results are shown in Supplementary Table S6; overall, these indicated no significant differences for most items across the different stages of the three different staging systems, although for the King’s staging system, most items showed significant worsening from stage 2 to stage 4.

#### EQ-5D-5L utility score

**Fig. 7:**
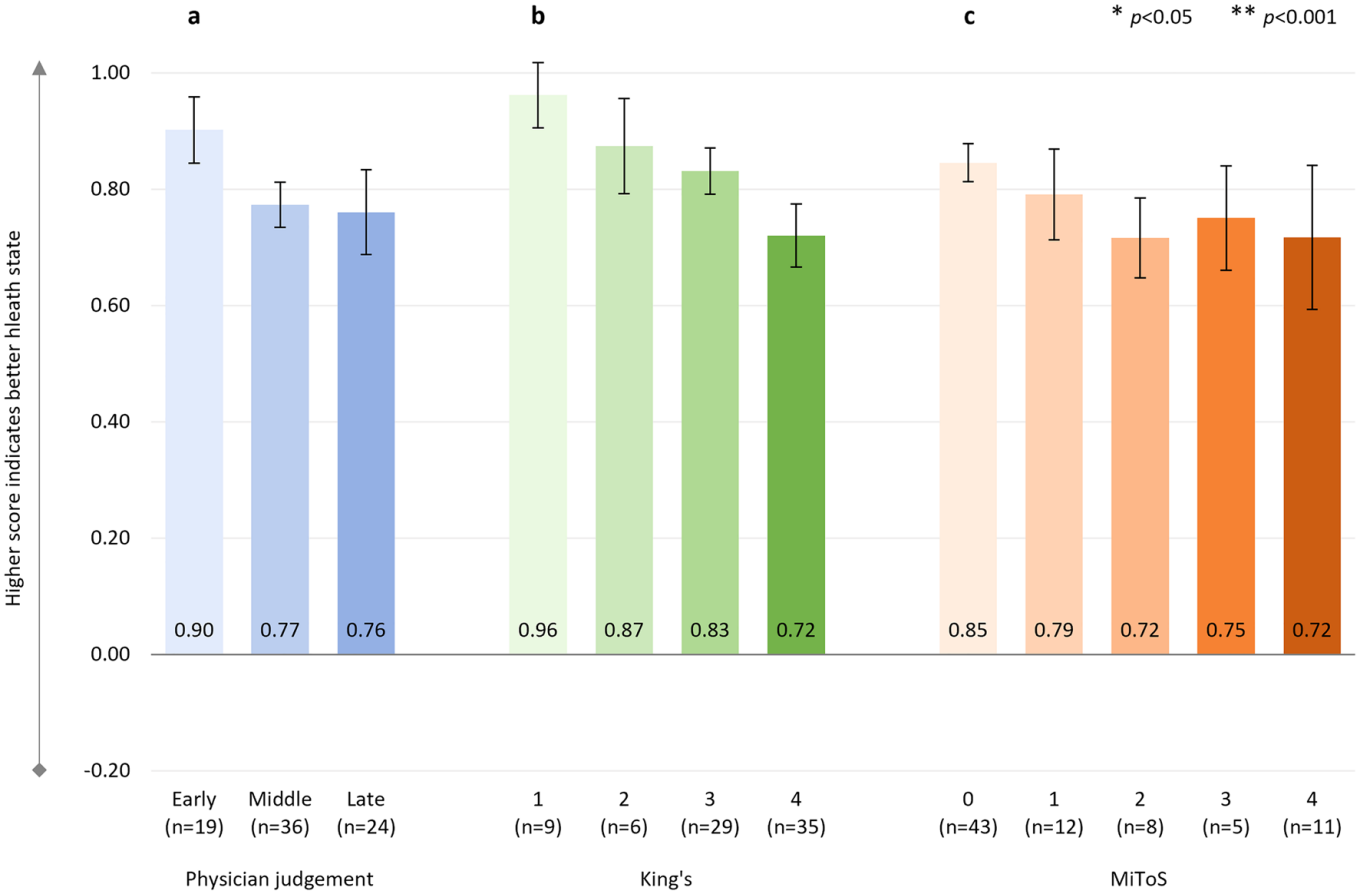
EQ-5D-5L utility score (cALS)

There were no significant correlations between cALS EQ-5D-5L utility scores and physician-judged staging (*r*^2^ = 0.051, *p* = 0.196, *n* = 79) or MiToS staging (*r*^2^ = 0.048, *p* = 0.303, *n* = 79) (Fig. 7a, b), although there was a significant negative correlation between EQ-5D-5L utility score and King’s staging, with a stepwise decline in health status (Fig. 7c).

Domain-level results are shown in Supplementary Table S7. While most individual domains for cALS showed no significant worsening over different stages regardless of the staging system, mobility significantly worsened across stages using the King’s staging system and a similar trend was observed for pain/discomfort.

#### EQ-5D-VAS

**Fig. 8:**
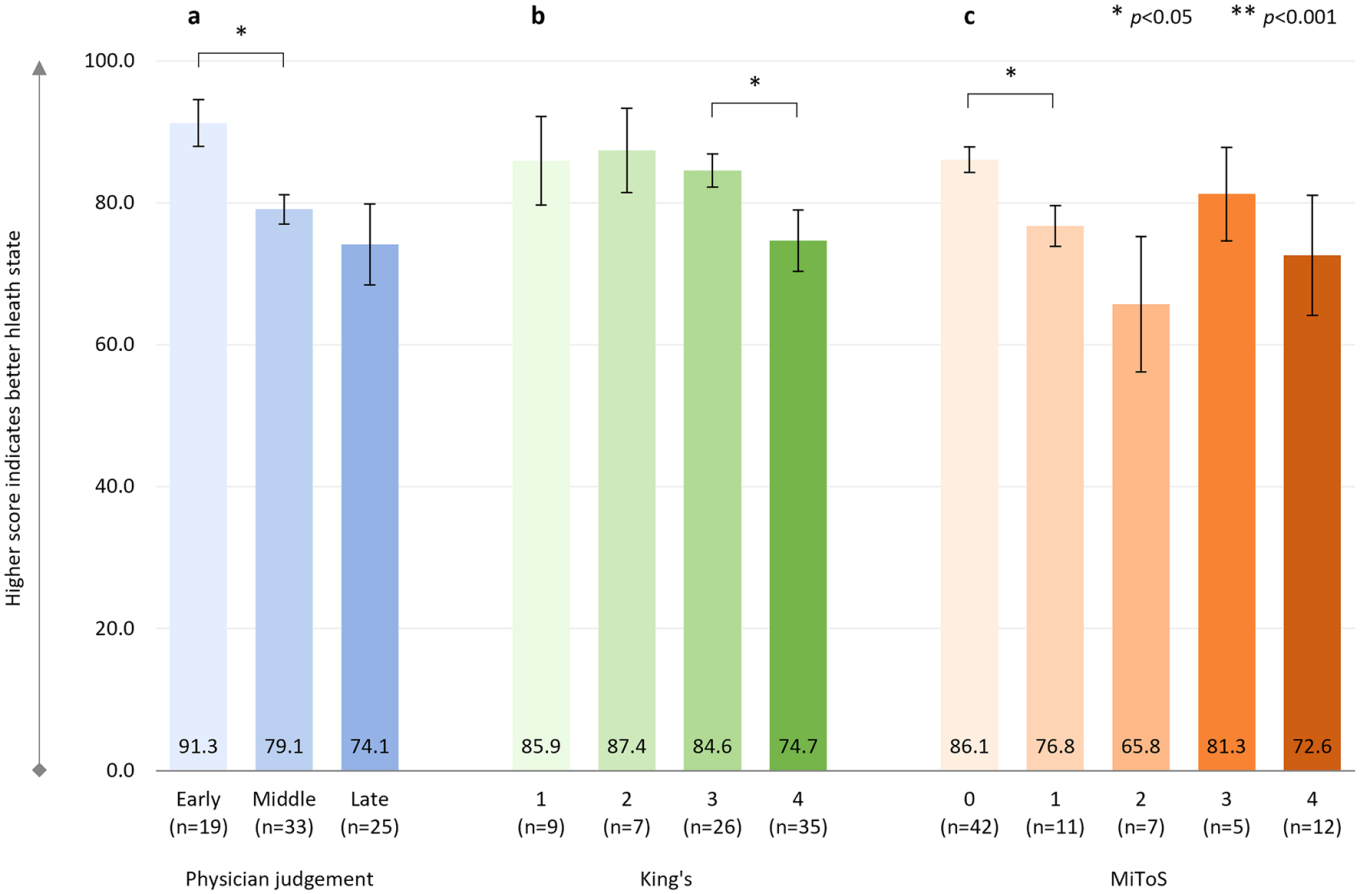
EQ-VAS score (cALS)

For cALS EQ-5D-VAS score, there was a significant positive correlation versus physician judgement: *r*^2^ = 0.134, *p* < 0.001 (*n* = 77), with cALS of ‘middle’ ALS stage patients rating their health state significantly lower than those of ‘early’ ALS stage patients (Fig. 8a). In addition, a significant non-linear correlation was observed between MiToS staging and EQ-5D-VAS score (*r*^2^ = 0.155, *p* = 0.003 [*n* = 77]; Fig. 8b), but there was no significant correlation between King’s staging and EQ-5D-VAS score (*r*^2^ = 0.109, *p* = 0.234 [*n* = 77]; Fig. 8c).

## Discussion

ALS is typically characterized by a rapid disease course, with pALS progressing from normal functioning to requiring assistance in basic functions and then death within 3–5 years. As confirmed by the present analysis, ALS has a substantial negative impact on QoL across multiple domains, such as mobility, self-care, emotional functioning, activity impairment, fatigue, and care partner burden. Our analysis showed that across all PRO measures assessed, pALS and cALS in this real-world study generally reported worse outcomes at later stages of the disease.

Across the King’s staging, the steepest decline in health status and HRQoL was observed between stages 3 and 4, which coincides with the introduction of feeding and/or ventilation assistance, due to nutritional and/or respiratory failure. In contrast, across the MiToS staging, the steepest decline in health status and HRQoL was observed between stages 0 and 1 and/or between stages 1 and 2, following the loss of independent function in one or two functional domains, respectively. From this stage onwards, pALS EQ-5D-5L utility scores indicated an HRQoL state close to or worse than death. For MiToS, where complete loss of function in an additional domain is required to progress to the subsequent stage, a steeper health status and HRQoL decline was observed across stages than for King’s. Across physician judgment staging, the steepest decline in HRQoL varied by PRO and was observed between the ‘middle’ and ‘late’ ALS stages for EQ-5D-5L, and between the ‘early’ and ‘middle’ stages for ALSAQ-5. This observed difference may reflect the domains covered by the two tools (ALSAQ-5: physical mobility, activities of daily living, eating/drinking, communication, and emotional functioning; EQ-5D: mobility, usual activities, self-care, pain/discomfort, and anxiety/depression). The ALSAQ-5 is condition-specific and may therefore be more sensitive to earlier changes in HRQoL, whereas the EQ-5D-5L may be more sensitive in areas that, while not specific to ALS, are important to pALS, especially in later stages of the disease. In the current study we observed worsening of pALS EQ-5D-5L pain/discomfort across King's and physician-judgement staging, and also worsening at MiToS stages 2/3 versus earlier stages. Hence this appears to be a domain that is not only important to pALS, but, unlike mobility, self-care, and usual activities, should be treatable, offering the potential to improve patient outcomes in later stages of the disease. Effective pain treatment can improve pALS and cALS’ QoL, even in the absence of a disease cure [43].

This also applies to the domain of anxiety/depression on the EQ-5D-5L and the hopelessness item of ALSAQ-5, with relatively few pALS reporting that they were not anxious or depressed, and with pALS scoring highly on the ‘I have felt hopeless about the future’ item of ALSAQ-5 across all stages of disease and regardless of the staging system, suggesting that pALS’ mental health/well-being should be addressed from the early stages of the disease. Körner and colleagues [44] reported that depressive symptoms had a strong influence on QoL in their cohort of German pALS and highlighted the importance of regular evaluation, timely diagnosis, and treatment of these symptoms.

In the recent systematic review of QoL deterioration in ALS by Forsythe and colleagues [11], a severe decline in health status and HRQoL was found to correlate with increased disease severity, with EQ-5D utility values ranging from 0.79 to 0.65 at stage 1, 0.67–0.53 at stage 2, 0.71–0.35 at stage 3, and 0.50–0.12 at stage 4 on the King’s staging system [30, 45–47]. The results of the present investigation, with adjusted mean EQ-5D-5L utility scores ranging from 0.65 at King’s stage 1 to 0.11 at stage 4, are therefore in line with previous findings of QoL changes in pALS and are well below general population norms in the UK and other countries (0.83–0.92) [48]. They are also considerably below the meta-analytic/pooled utility scores reported in patients with other chronic conditions such as diabetes mellitus (random-effect model, 0.83; fixed-effect model, 0.93), neoplasms (0.75; 0.80), cardiovascular disease (0.77; 0.76), multiple sclerosis (0.56; 0.67), chronic obstructive pulmonary disease (0.75; 0.76), HIV infection (0.84; 0.81), and chronic kidney disease (0.70; 0.76) [49]. Furthermore, the current adjusted mean EQ-5D-5L utility values ranged from 0.53 at stage 0 on the MiToS staging system to − 0.10 at stage 4, which were considerably lower than the mean values previously reported by Moore and colleagues in respondents with motor neurone disease in the UK (0.71 at stage 0; 0.25 at stage 4) [30]. However, it should be noted that Moore and colleagues calculated utility values using UK 5L value sets [28], whereas for the present investigation cross-walked 3L UK value sets were used, according to current NICE guidelines [32–34]. Additionally, Moore and colleagues reported data from the UK only, whereas the present study pooled data from six countries. The mean EQ-5D-VAS scores in this study were also lower at each disease stage than those reported by Moore et al., whether using the King’s or the MiToS staging systems [30].

Finally, similar results obtained using pALS-reported and cALS-reported EQ-5D-5L and EQ-5D-VAS scores suggest that a proxy care partner score is a valid alternative when the person’s own perspective of his/her general health is missing. The present findings are also interesting because patient-perceived QoL often differs from QoL perceptions/ratings reported by care partners, the general public, and healthcare professionals, who may considerably underestimate pALS’ QoL [14, 50, 51].

Although not all differences were significant, ALSAQ-5 scores showed a stepwise increase in HRQoL impairment at each progressing stage of the disease regardless of the staging system. Similar findings were reported by Peseschkian and colleagues [52] using the King’s staging system in pALS in Germany, up to and including stage 4a [52].

Nevertheless, as already mentioned, several previous investigations showed satisfactory QoL, as reported by pALS, in both earlier and later stages of the disease, or despite progressing physical decline [12–15, 51]. This may be due to a combination of patient-related factors, such as psychosocial support, successful coping strategies, spirituality and religiosity, early palliative care provision, cultural background [24, 53, 54], or methodological factors, such as the specific instruments used to assess QoL in different studies.

The steepest increase in the severity of fatigue, as assessed by the FSS, was seen between the ‘early’ and ‘middle’ ALS stages when using physician judgment staging, and between stages 0 and 1 when using MiToS staging (as well as between stages 3 and 4); more subtle differences were observed with King’s staging, although mean FSS scores were clinically significant at all stages (i.e., above the cut-off of total score of > 40). By later stage disease, high levels of fatigue were reported, with scores of 53, 54, 54 for physician judgment ‘late’ stage, King’s stage 4 and MiToS stage 3, respectively (out of a score range of 0–63). These results indicate that high levels of fatigue are present in ALS, even in the early stages of the disease, and that the change/escalation of fatigue earlier in the disease course may be perceived as more impairing/impactful on daily function by pALS. Of note, pALS self-reported fatigue among their three most disabling symptoms even at late stages of the disease, when one might expect other symptoms or complications to become and/or be perceived as more disabling. A recent systematic review and meta-analysis confirmed that fatigue was a common (the pooled frequency of fatigue across all studies was 48%) and clinically significant symptom in pALS, which was associated with the severity of disease, as measured by the ALSFRS-R, highlighting the importance of early assessment and management of fatigue in pALS [55].

With regards to activity impairment score on the WPAI, similar findings were observed, with the steepest increase in activity impairment being evident between the ‘early’ and ‘middle’ ALS stages when using physician judgment staging, between stages 0 and 1 when using MiToS staging, and between stages 1 and 2 when using King’s staging. By later stage disease, substantial activity impairment was reported, with 65%, 69%, 88% and 70% impairment for physician judgment stage ‘late’, King’s stage 4 and MiToS stages 2 and 3, respectively.

As patients' disease states progressed, their cALS also experienced a decline in health status and HRQoL, as assessed by the EQ-5D-5L and the EQ-5D-VAS, and an increase in burden, as assessed by the ZBI-12, although none of the correlations between ZBI-12 and the different staging systems were statistically significant. Nonetheless, cALS-reported burden started high at MiToS stage 0 (above the cut-off of ≥ 17, which indicates a high burden) and remained high using this staging system. High burden was also reported for physician judgement stages ‘mid’ and ‘late’ and King’s stages 3 and 4. Brizzi and colleagues [56] recently found that cALS in the US self-reported significantly higher stress levels than pALS reported for themselves; in addition, 35% of cALS reported experiencing a devastating or near devastating financial impact of ALS and 64% felt that their own health had worsened. A previous systematic review found that a higher cALS burden was associated with greater patient behavioral and physical impairment, as well as increased depressive symptoms [57]. Also, a recent study in Poland and Germany revealed that both QoL and mood were significantly lower in cALS who were more burdened with the functional care of pALS, highlighting the need for stronger social- and healthcare-system support to family members of pALS who carry the main burden of personal care [58]. Similarly, Linse and colleagues [59] found that cALS burden in Germany was at least partly dependent on modifiable aspects of socio-medical care and that unmet cALS needs were associated with increased health problems in the cALS themselves.

The worsening in cALS’ EQ-5D-5L score across King’s staging observed in the current study was primarily driven by the mobility and pain/discomfort domains, which may reflect the physical exertion resulting from caring.

Across a few outcome measures, we saw a gradual worsening across stages 0–2 or 0–3 of the MiToS staging system, before seeing a slight improvement again at stage 3 or 4, respectively, depending on the outcome measure. This pattern may have occurred by chance alone, as the group sizes for the latter MiToS stages were small. Alternatively, it is possible that pALS expectations regarding QoL may shift over the course of the disease, with evolving expectations or perceptions of how ‘usual activities’ are defined. Additionally, it may be that pALS are receiving more professional assistance with daily tasks in the later stages of the disease, which may impact QoL ratings. Indeed, throughout the disease process, pALS may experience shifting expectations and reprioritization of factors that contribute most to their QoL, from those dependent on physical function to factors such as family, friends, and spiritual support [50].

Stage 3 MiToS represent a considerable loss of function and stage 4 MiToS represents a near total loss of function and being wheelchair-/bed-bound may impact the level of fatigue at later stages of disease. Further, implementation of end-of-life care may result in cALS reporting slightly less burden as a result of greater involvement of professional caregivers.

Overall, the current findings demonstrate the impact of ALS disease progression on HRQoL in the real-world across multiple domains (mobility, self-care, emotional functioning, activity impairment, fatigue, caregiver burden) and highlight the potential impact of delaying disease progression in ALS. Detailing the experiences of pALS over the disease course may help those living with the disease, and their families, plan for future needs.

The present study has several limitations: the DSP is not based on a truly random sample of neurologists and pALS; while minimal inclusion criteria governed the selection of the participating neurologists, participation was influenced by the willingness to complete the survey. In cases where cALS helped pALS complete their written form, the responses may have been influenced by the care partner involvement. Additionally, the COVID-19 pandemic may have played a role in the recruitment of participants, although the impact of the pandemic is expected to have been minimal due to the nature and severity of the condition, and the associated need for frequent routine medical care; nevertheless, during the first wave of the pandemic pALS did experience a lack of face-to-face contact with healthcare professionals and delays to evaluation and treatment [60].

Also, the DSP is a cross-sectional survey; therefore, different individuals (with different value judgements) made up the groups for each disease stage rather than a fixed cohort of pALS and cALS being followed over time and the survey findings cannot be used to demonstrate cause and effect; however, identification of significant associations/correlations is possible. Lastly, while the DSP methodology requiring neurologists to consult with pALS prior to completing/accessing the medical records may reduce the risk of recall bias, this cannot be completely eliminated.

Methodological strengths of the study include use of three different ALS staging systems and collection of pALS and cALS perspectives; use of a real-world study design that enabled the capture of HRQoL data for pALS; pALS being identified by physician-confirmed diagnosis (rather than self-report); the ability to link data from up to three sources (physician, self, care partner) for each pALS; and data being collected independently of any therapeutic intervention or clinical management strategy.

## Conclusions

This real-world international survey offers insight into the impact of ALS disease progression on health status and HRQoL of pALS and cALS, with worse outcomes (frequently above thresholds for clinical meaningfulness/high burden) being reported at later stages of the disease, with EQ-5D-5L health status being well below that of the general population and considerably below that of other chronic conditions. The findings highlight an unmet need in this population for strategies to maximise QoL despite disease progression, and the potential importance of slowing disease progression, allowing pALS to remain at earlier stages of disease longer. Additionally, greater recognition of the burden associated with symptoms such as pain and fatigue, and attempts to treat these where present, may lead to improved QoL for both pALS and cALS.

### Supplementary Information

Below is the link to the electronic supplementary material.

Supplementary file1 (PDF 373 kb)

## Data Availability

All data that support the findings of this study are the intellectual property of Adelphi Real World. All requests for access should be addressed directly to Jennifer Mellor at jennifer.mellor@adelphigroup.com.
