# Supplementary material for: Health-related quality of life across disease stages in patients with amyotrophic lateral sclerosis: results from a real-world survey
Source: J Neurol. 2024 Jan 11;271(5):2390–404. doi: 10.1007/s00415-023-12141-y (PMC11055770; doi:10.1007/s00415-023-12141-y)
Supplement: Supplementary file 1 — Supplementary file1 (PDF 373 kb) [file 415_2023_12141_MOESM1_ESM.pdf]

Health-related quality of life across disease stages in patients with amyotrophic lateral sclerosis: Results from a real-world survey

Stenson K,[1] Fecteau TE,[1] O'Callaghan L,[1*] Bryden P,[1] Mellor J,[2] Wright J,[2] Earl L,[2] Thomas O,[2] Iqbal H,[2] Barlow S,[2] Parvanta S[3]

[1]Biogen, Cambridge, MA, USA; [2]Adelphi Real World, Bollington, UK; [3]The ALS Association, Arlington, VA, USA

**Present address: Sage Therapeutics*

Corresponding author: Katie Stenson; **Email:** katie.stenson@biogen.com

Supplementary Table 1 Comparison of physician judged ALS stage with King's and MiToS staging systems

	Physician judgement		
	**Early stage**	**Middle stage**	**Late stage**
**King's stage, n (%)**			
n	*61*	*76*	*32*
Stage 1	22 (36.1)	7 (9.2)	0 (0.0)
Stage 2	16 (26.2)	11 (14.5)	0 (0.0)
Stage 3	22 (36.1)	36 (47.4)	3 (9.4)
Stage 4a/b	1 (1.6)	22 (28.9)	29 (90.6)
**MiToS stage, n (%)**			
n	*64*	*76*	*32*
Stage 0	64 (100.0)	57 (75.0)	1 (3.1)
Stage 1	0 (0.0)	13 (17.1)	5 (15.6)
Stage 2	0 (0.0)	5 (6.6)	5 (15.6)
Stage 3	0 (0.0)	1 (1.3)	7 (21.9)
Stage 4	0 (0.0)	0 (0.0)	14 (43.8)

MiToS: Milano-Torino Staging

Supplementary Table 2 Comparison of patient-reported versus care partner-reported (proxy) EQ-5D-5L utility and EQ-5D-VAS scores

	EQ-5D-5L (n=42)			EQ-5D-VAS (n=41)		
	**Patient-reported**	**Proxy**	**p-value**	**Patient-reported**	**Proxy**	**p-value**
**Mean (SD)**	0.399 (0.282)	0.370 (0.271)	0.176	43.6 (21.5)	43.0 (20.8)	0.799
**Range**	-0.200, 0.891	-0.200, 0.891		5.0, 84.0	5.0, 95.0	

EQ-5D-5L: EuroQol (European Quality of Life) Five Dimension Five Level Scale; EQ-5D-VAS: EuroQol (European Quality of Life) Five Dimension Five Level Scale-Visual Analogue Scale; SD: standard deviation

Supplementary Table 3 Item level responses to the EQ-5D-5L for pALS by physician judgement, King's and MiToS staging

	King's staging					MiToS						Physician judgement			
	Stage 1 (n=29)	Stage 2 (n=27)	Stage 3 (n=56)	Stage 4 (n=50)	*p*-value	Stage 0 (n=116)	Stage 1 (n=17)	Stage 2 (n=10)	Stage 3 (n=8)	Stage 4 (n=14)	*p*-value	Early (n=61)	Middle (n=72)	Late (n=32)	*p*-value
**Mobility, n (%)**															
No problems walking about	15 (51.7)	5 (18.5)	1 (1.8)	1 (2.0)		21 (18.1)	0 (0.0)	0 (0.0)	0 (0.0)	1 (7.1)		15 (24.6)	6 (8.3)	1 (3.1)	
Slight problems walking about	6 (20.7)	10 (37.0)	19 (33.9)	5 (10.0)		40 (34.5)	1 (5.9)	0 (0.0)	1 (12.5)	1 (7.1)		24 (39.3)	17 (23.6)	2 (6.3)	
Moderate problems walking about	3 (10.3)	9 (33.3)	22 (39.3)	11 (22.0)	<0.001	38 (32.8)	5 (29.4)	1 (10.0)	1 (12.5)	0 (0.0)	<0.001	16 (26.2)	27 (37.5)	2 (6.3)	<0.001
Severe problems walking about	3 (10.3)	1 (3.7)	12 (21.4)	13 (26.0)		16 (13.8)	6 (35.3)	5 (50.0)	2 (25.0)	0 (0.0)		6 (9.8)	16 (22.2)	7 (21.9)	
Unable to walk about	2 (6.9)	2 (7.4)	2 (3.6)	20 (40.0)		1 (0.9)	5 (29.4)	4 (40.0)	4 (50.0)	12 (85.7)		0 (0.0)	6 (8.3)	20 (62.5)	
**Self-Care, n (%)**															
No problems washing or dressing myself	16 (55.2)	7 (25.9)	8 (14.3)	2 (4.0)		34 (29.3)	1 (5.9)	0 (0.0)	0 (0.0)	1 (7.1)		26 (42.6)	9 (12.5)	1 (3.1)	
Slight problems washing or dressing myself	5 (17.2)	13 (48.2)	22 (39.3)	7 (14.0)		44 (37.9)	2 (11.8)	0 (0.0)	1 (12.5)	0 (0.0)		22 (36.1)	22 (30.6)	3 (9.4)	
Moderate problems washing or dressing myself	8 (27.6)	5 (18.5)	17 (30.4)	11 (22.0)	<0.001	30 (25.9)	7 (41.2)	2 (20.0)	2 (25.0)	0 (0.0)	<0.001	11 (18.0)	27 (37.5)	3 (9.4)	<0.001
Severe problems washing or dressing myself	0 (0.0)	0 (0.0)	8 (14.3)	9 (18.0)		7 (6.0)	5 (29.4)	4 (40.0)	1 (12.5)	0 (0.0)		2 (3.3)	11 (15.3)	4 (12.5)	
Unable to wash or dress myself	0 (0.0)	2 (7.4)	1 (1.8)	21 (42.0)		1 (0.9)	2 (11.8)	4 (40.0)	4 (50.0)	13 (92.9)		0 (0.0)	3 (4.2)	21 (65.6)	
**Usual Activities, n (%)**															
No problems doing my usual activities	13 (44.8)	2 (7.4)	6 (10.7)	1 (2.0)		22 (19.0)	1 (5.9)	0 (0.0)	0 (0.0)	1 (7.1)		18 (29.5)	5 (6.9)	1 (3.1)	
Slight problems doing my usual activities	6 (20.7)	13 (48.2)	19 (33.9)	3 (6.0)		40 (34.5)	2 (11.8)	0 (0.0)	0 (0.0)	0 (0.0)		25 (41.0)	17 (23.6)	0 (0.0)	
Moderate problems doing my usual activities	6 (20.7)	8 (29.6)	18 (32.1)	13 (26.0)	<0.001	36 (31.0)	5 (29.4)	2 (20.0)	2 (25.0)	0 (0.0)	<0.001	14 (23.0)	29 (40.3)	2 (6.3)	<0.001
Severe problems doing my usual activities	3 (10.3)	4 (14.8)	12 (21.4)	13 (26.0)		16 (13.8)	9 (52.9)	4 (40.0)	2 (25.0)	1 (7.1)		4 (6.6)	19 (26.4)	9 (28.1)	
Unable to do my usual activities	1 (3.5)	0 (0.0)	1 (1.8)	20 (40.0)		2 (1.7)	0 (0.0)	4 (40.0)	4 (50.0)	12 (85.7)		0 (0.0)	2 (2.8)	20 (62.5)	
**Pain/Discomfort, n (%)**															
No pain or discomfort	20 (69.0)	9 (33.3)	17 (30.4)	8 (16.0)		48 (41.4)	4 (23.5)	1 (10.0)	0 (0.0)	4 (28.6)		31 (50.8)	20 (27.8)	6 (18.8)	
Slight pain or discomfort	5 (17.2)	13 (48.2)	21 (37.5)	13 (26.0)		39 (33.6)	4 (23.5)	5 (50.0)	2 (25.0)	2 (14.3)		20 (32.8)	25 (34.7)	7 (21.9)	
Moderate pain or discomfort	3 (10.3)	5 (18.5)	14 (25.0)	19 (38.0)	<0.001	23 (19.8)	8 (47.1)	2 (20.0)	3 (37.5)	5 (35.7)	0.001	9 (14.8)	22 (30.6)	10 (31.3)	<0.001
Severe pain or discomfort	1 (3.5)	0 (0.0)	4 (7.1)	6 (12.0)		6 (5.2)	1 (5.9)	1 (10.0)	2 (25.0)	1 (7.1)		1 (1.6)	5 (6.9)	5 (15.6)	
Extreme pain or discomfort	0 (0.0)	0 (0.0)	0 (0.0)	4 (8.0)		0 (0.0)	0 (0.0)	1 (10.0)	1 (12.5)	2 (14.3)		0 (0.0)	0 (0.0)	4 (12.5)	
**Anxiety/Depression, n (%)**															
Not anxious or depressed	3 (10.3)	3 (11.1)	6 (10.7)	4 (8.0)		10 (8.6)	2 (11.8)	0 (0.0)	1 (12.5)	3 (21.4)		7 (11.5)	5 (6.9)	4 (12.5)	
Slightly anxious or depressed	14 (48.3)	9 (33.3)	10 (17.9)	11 (22.0)		35 (30.2)	8 (47.1)	1 (10.0)	1 (12.5)	1 (7.1)		21 (34.4)	21 (29.2)	4 (12.5)	
Moderately anxious or depressed	9 (31.0)	8 (29.6)	25 (44.6)	19 (38.0)	0.050	43 (37.1)	5 (29.4)	3 (30.0)	4 (50.0)	7 (50.0)	0.034	22 (36.1)	27 (37.5)	13 (40.6)	0.106
Severely anxious or depressed	2 (6.9)	5 (18.5)	13 (23.2)	8 (16.0)		22 (19.0)	2 (11.8)	3 (30.0)	1 (12.5)	0 (0.0)		7 (11.5)	16 (22.2)	5 (15.6)	
Extremely anxious or depressed	1 (3.5)	2 (7.4)	2 (3.6)	8 (16.0)		6 (5.2)	0 (0.0)	3 (30.0)	1 (12.5)	3 (21.4)		4 (6.6)	3 (4.2)	6 (18.8)	

Supplementary Table 4 Item level results for the ALSAQ-5 by physician judgement, King's and MiToS staging

	**King's staging**					**MiToS**						**Physician judgement**			
	Stage 1 (n=25)	Stage 2 (n=26)	Stage 3 (n=50)	Stage 4 (n=30)	*p*-value	Stage 0 (n=110)	Stage 1 (n=13)	Stage 2 (n=4)	Stage 3 (n=5)	Stage 4 (n=0)	*p*-value	Early (n=61)	Middle (n=64)	Late (n=9)	*p*-value
**I have found it difficult to stand up**															
Mean	1.9	2.6	3.1	3.3	<0.001	2.7	3.5	3.9	3.2	-	<0.001	2.4	3.1	2.9	<0.001
SE	0.2	0.2	0.1	0.2		0.1	0.1	0.3	0.7	-		0.1	0.1	0.5	
**I have had difficulty using my arms and hands**															
Mean	2.1	3.0	3.0	3.4	<0.001	2.8	2.9	3.6	3.6	-	0.002	2.6	3.1	3.1	0.028
SE	0.2	0.1	0.1	0.2		0.1	0.3	0.2	0.3	-		0.2	0.1	0.6	
**I have had difficulty eating solid food**															
Mean	1.4	1.9	2.7	2.9	<0.001	2.2	2.2	4.0	2.3	-	<0.001	1.9	2.6	2.6	<0.001
SE	0.1	0.2	0.2	0.2		0.1	0.4	0.2	1.0	-		0.1	0.2	0.9	
**I have felt that my speech has not been easy**															
Mean	1.6	1.8	2.8	2.9	<0.001	2.3	2.3	3.7	0.9	-	<0.001	2.0	2.6	2.7	0.002
SE	0.1	0.2	0.1	0.2		0.1	0.3	0.2	0.1	-		0.1	0.2	0.7	
**I have felt hopeless about the future**															
Mean	2.5	2.9	3.3	3.4	0.002	3.0	3.3	4.1	3.2	-	<0.001	2.8	3.3	3.2	0.064
SE	0.2	0.2	0.1	0.2		0.1	0.3	0.2	0.1	-		0.2	0.1	0.5	

Supplementary Table 5 Item level results for the FSS by physician judgement, King's and MiToS staging

	King's staging					MiToS						Physician judgement			
	Stage 1 (n=25)	Stage 2 (n=26)	Stage 3 (n=49)	Stage 4 (n=29)	*p*-value	Stage 0 (n=108)	Stage 1 (n=13)	Stage 2 (n=4)	Stage 3 (n=5)	Stage 4 (n=0)	*p*-value	Early (n=60)	Middle (n=63)	Late (n=8)	*p*-value
**My motivation is lower when I am fatigued**															
Mean	5.0	5.7	5.7	6.4	<0.001	5.6	6.0	5.6	6.8	-	<0.001	5.3	6.1	6.1	0.007
SE	0.3	0.3	0.2	0.1		0.1	0.3	0.5	0.2	-		0.2	0.1	0.3	
**Exercise makes me fatigued**															
Mean	5.0	5.7	5.8	6.0	0.159	5.5	6.3	6.0	6.2	-	0.157	5.2	6.0	6.2	0.023
SE	0.4	0.3	0.2	0.2		0.1	0.4	0.6	0.7	-		0.2	0.2	0.5	
**I am easily fatigued**															
Mean	4.7	5.6	5.7	6.0	0.029	5.4	6.3	6.3	5.9	-	0.025	5.1	5.9	6.0	0.015
SE	0.4	0.3	0.2	0.2		0.1	0.3	0.5	0.9	-		0.2	0.2	0.6	
**Fatigue interferes with my ability to function physically**															
Mean	4.5	5.6	6.0	6.0	0.002	5.4	6.6	6.6	5.8	-	<0.001	5.1	6.1	6.0	<0.001
SE	0.4	0.3	0.2	0.2		0.2	0.2	0.3	0.7	-		0.2	0.2	0.5	
**Fatigue frequently causes problems for me**															
Mean	3.9	5.2	5.6	6.3	<0.001	5.1	6.2	6.4	6.2	-	0.007	4.6	5.9	6.1	<0.001
SE	0.4	0.4	0.2	0.2		0.2	0.3	0.5	0.7	-		0.3	0.2	0.5	
**My fatigue prevents sustained physical functioning**															
Mean	4.3	5.3	5.8	5.9	0.005	5.3	6.2	6.3	5.7	-	0.009	4.9	5.8	5.7	0.003
SE	0.4	0.3	0.2	0.3		0.2	0.3	0.2	1.1	-		0.2	0.2	0.7	
**Fatigue interferes with carrying out certain duties and responsibilities**															
Mean	4.4	5.4	5.6	5.9	0.016	5.2	6.1	6.1	5.5	-	0.060	4.8	5.8	5.8	0.004
SE	0.4	0.4	0.2	0.3		0.2	0.4	0.4	1.0	-		0.3	0.2	0.7	
**Fatigue is among my 3 most disabling symptoms**															
Mean	3.9	4.8	5.5	5.7	<0.001	5.0	5.8	5.3	5.4	-	0.239	4.4	5.7	4.9	<0.001
SE	0.3	0.4	0.2	0.3		0.2	0.4	0.6	1.0	-		0.2	0.2	0.8	
**Fatigue interferes with my work, family, or social life**															
Mean	3.9	4.9	5.5	5.9	<0.001	4.9	6.1	6.2	5.7	-	0.006	4.4	5.8	5.7	<0.001
SE	0.3	0.4	0.3	0.3		0.2	0.4	0.5	1.1	-		0.3	0.2	0.7	

Supplementary Table 6 Item level results for the ZBI-12 by physician judgement, King's and MiToS staging

	King's staging					MiToS						Physician judgement			
	Stage 1 (n=9)	Stage 2 (n=6)	Stage 3 (n=29)	Stage 4 (n=36)	*p*-value	Stage 0 (n=44)	Stage 1 (n=11)	Stage 2 (n=8)	Stage 3 (n=5)	Stage 4 (n=12)	*p*-value	Early (n=20)	Middle (n=35)	Late (n=25)	*p*-value
**Do you feel that because of the time you spend with your relative that you don't have enough time for yourself?**															
Mean	2.0	2.5	3.2	3.4	0.017	2.8	3.4	3.8	3.5	3.1	0.175	2.4	3.2	3.4	0.128
SE	0.4	0.6	0.2	0.2		0.2	0.3	0.4	0.6	0.4		0.4	0.1	0.3	
**Do you feel stressed between caring for your relative and trying to meet other responsibilities for your family or work?**															
Mean	2.3	2.5	3.2	3.4	0.063	2.9	2.7	4.1	3.5	3.4	0.119	2.3	3.2	3.7	0.014
SE	0.4	0.5	0.2	0.3		0.2	0.3	0.4	0.5	0.5		0.3	0.2	0.3	
**Do you feel angry when you are around the relative?**															
Mean	1.7	1.7	2.6	2.3	0.083	2.3	2.2	2.3	1.9	2.4	0.793	2.1	2.4	2.3	0.683
SE	0.4	0.3	0.3	0.2		0.3	0.2	0.4	0.5	0.4		0.4	0.2	0.2	
**Do you feel that your relative currently affects your relationships with other family members or friends in a negative way?**															
Mean	1.8	1.3	2.3	2.7	0.012	2.3	1.7	2.7	2.1	3.0	0.119	1.8	2.3	2.9	0.052
SE	0.5	0.4	0.3	0.3		0.2	0.3	0.5	0.4	0.5		0.4	0.2	0.3	
**Do you feel strained when you are around your relative?**															
Mean	1.9	1.6	2.7	2.9	0.040	2.5	2.5	3.4	2.5	2.9	0.249	2.2	2.7	2.9	0.336
SE	0.4	0.6	0.2	0.2		0.2	0.4	0.3	0.5	0.5		0.4	0.2	0.3	
**Do you feel that your health has suffered because of your involvement with your relative?**															
Mean	1.8	1.7	2.4	2.7	0.084	2.3	2.2	3.1	2.5	2.7	0.530	2.0	2.5	2.8	0.232
SE	0.5	0.4	0.3	0.3		0.3	0.3	0.5	0.6	0.5		0.4	0.3	0.3	
**Do you feel that you don't have as much privacy as you would like because of your relative?**															
Mean	1.9	1.6	2.4	3.0	0.015	2.4	1.9	3.8	2.4	3.1	0.022	1.9	2.6	3.0	0.021
SE	0.4	0.5	0.3	0.3		0.2	0.3	0.5	0.6	0.4		0.3	0.3	0.3	
**Do you feel that your social life has suffered because you are caring for your relative?**															
Mean	2.0	1.7	2.8	3.2	0.029	2.7	2.2	3.9	2.8	3.2	0.015	2.2	2.9	3.2	0.060
SE	0.5	0.7	0.2	0.3		0.3	0.3	0.5	0.6	0.5		0.4	0.3	0.3	
**Do you feel that you have lost control of your life since your relative's illness?**															
Mean	1.8	1.5	2.7	2.9	0.024	2.6	1.8	3.3	2.2	3.1	0.043	2.2	2.7	2.9	0.396
SE	0.5	0.5	0.3	0.3		0.3	0.2	0.5	0.6	0.5		0.5	0.2	0.4	
**Do you feel uncertain about what to do about your relative?**															
Mean	2.4	2.0	3.2	3.0	<0.001	2.8	2.9	3.5	3.0	3.1	0.597	2.5	3.1	3.1	0.441
SE	0.5	0.3	0.2	0.2		0.3	0.3	0.3	0.6	0.5		0.5	0.2	0.3	
**Do you feel you should be doing more for your relative?**															
Mean	2.6	2.8	3.5	3.3	0.226	3.2	3.5	3.7	3.6	2.9	0.424	2.9	3.5	3.2	0.380
SE	0.5	0.7	0.2	0.2		0.3	0.2	0.3	0.3	0.5		0.5	0.2	0.3	
**Do you feel you could do a better job in caring for your relative?**															
Mean	2.5	3.2	3.3	3.0	0.179	3.1	3.3	3.3	3.3	2.7	0.733	2.9	3.3	3.0	0.272
SE	0.3	0.5	0.2	0.2		0.2	0.3	0.3	0.4	0.4		0.4	0.2	0.3	

Supplementary Table 7 Item level responses to the EQ-5D-5L for cALS by physician judgement, King's and MiToS staging

	King's staging					MiToS						Physician judgement			
	Stage 1 (n=9)	Stage 2 (n=7)	Stage 3 (n=30)	Stage 4 (n=35)	*p*-value	Stage 0 (n=45)	Stage 1 (n=12)	Stage 2 (n=8)	Stage 3 (n=5)	Stage 4 (n=11)	*p*-value	Early (n=21)	Middle (n=37)	Late (n=24)	*p*-value
**Mobility, n (%)**															
No problems walking about	9 (100.0)	7 (100.0)	27 (90.0)	21 (60.0)		39 (86.7)	10 (83.3)	5 (62.5)	2 (40.0)	8 (72.7)		19 (95.0)	28 (75.7)	17 (70.8)	
Slight problems walking about	0 (0.0)	0 (0.0)	1 (3.3)	6 (17.1)		2 (4.4)	1 (8.3)	2 (25.0)	1 (20.0)	1 (9.1)		0 (0.0)	3 (8.1)	4 (16.7)	
Moderate problems walking about	0 (0.0)	0 (0.0)	2 (6.7)	3 (8.6)	0.003	4 (8.9)	0 (0.0)	0 (0.0)	1 (20.0)	0 (0.0)	0.099	1 (5.0)	3 (8.1)	1 (4.2)	0.133
Severe problems walking about	0 (0.0)	0 (0.0)	0 (0.0)	3 (8.6)		0 (0.0)	0 (0.0)	1 (12.5)	1 (20.0)	1 (9.1)		0 (0.0)	2 (5.4)	1 (4.2)	
Unable to walk about	0 (0.0)	0 (0.0)	0 (0.0)	2 (5.7)		0 (0.0)	1 (8.3)	0 (0.0)	0 (0.0)	1 (9.1)		0 (0.0)	1 (2.7)	1 (4.2)	
**Self-Care, n (%)**															
No problems washing or dressing myself	9 (100.0)	7 (100.0)	28 (93.3)	29 (82.9)		42 (93.3)	11 (91.7)	7 (87.5)	4 (80.0)	9 (81.8)		19 (95.0)	33 (89.2)	21 (87.5)	
Slight problems washing or dressing myself	0 (0.0)	0 (0.0)	0 (0.0)	0 (0.0)		0 (0.0)	0 (0.0)	0 (0.0)	0 (0.0)	0 (0.0)		0 (0.0)	0 (0.0)	0 (0.0)	
Moderate problems washing or dressing myself	0 (0.0)	0 (0.0)	2 (6.7)	2 (5.7)	0.226	3 (6.7)	0 (0.0)	0 (0.0)	1 (20.0)	0 (0.0)	0.698	1 (5.0)	2 (5.4)	1 (4.2)	0.661
Severe problems washing or dressing myself	0 (0.0)	0 (0.0)	0 (0.0)	2 (5.7)		0 (0.0)	0 (0.0)	1 (12.5)	0 (0.0)	1 (9.1)		0 (0.0)	1 (2.7)	1 (4.2)	
Unable to wash or dress myself	0 (0.0)	0 (0.0)	0 (0.0)	2 (5.7)		0 (0.0)	1 (8.3)	0 (0.0)	0 (0.0)	1 (9.1)		0 (0.0)	1 (2.7)	1 (4.2)	
**Usual Activities, n (%)**															
No problems doing my usual activities	8 (88.9)	7 (100.0)	25 (83.3)	26 (74.3)		39 (86.7)	10 (83.3)	5 (62.5)	3 (60.0)	9 (81.8)		19 (95.0)	28 (75.7)	19 (79.2)	
Slight problems doing my usual activities	1 (11.1)	0 (0.0)	4 (13.3)	3 (8.6)		4 (8.9)	1 (8.3)	2 (25.0)	1 (20.0)	0 (0.0)		1 (5.0)	5 (13.5)	2 (8.3)	
Moderate problems doing my usual activities	0 (0.0)	0 (0.0)	1 (3.3)	3 (8.6)	0.301	2 (4.4)	0 (0.0)	1 (12.5)	1 (20.0)	0 (0.0)	0.405	0 (0.0)	3 (8.1)	1 (4.2)	0.182
Severe problems doing my usual activities	0 (0.0)	0 (0.0)	0 (0.0)	2 (5.7)		0 (0.0)	0 (0.0)	0 (0.0)	0 (0.0)	2 (18.2)		0 (0.0)	0 (0.0)	2 (8.3)	
Unable to do my usual activities	0 (0.0)	0 (0.0)	0 (0.0)	1 (2.9)		0 (0.0)	1 (8.3)	0 (0.0)	0 (0.0)	0 (0.0)		0 (0.0)	1 (2.7)	0 (0.0)	
**Pain/Discomfort, n (%)**															
No pain or discomfort	8 (88.9)	6 (85.7)	20 (66.7)	17 (48.6)		31 (68.9)	8 (66.7)	3 (37.5)	2 (40.0)	7 (63.6)		16 (80.0)	20 (54.1)	15 (62.5)	
Slight pain or discomfort	0 (0.0)	1 (14.3)	8 (26.7)	10 (28.6)		10 (22.2)	2 (16.7)	3 (37.5)	2 (40.0)	2 (18.2)		3 (15.0)	12 (32.4)	4 (16.7)	
Moderate pain or discomfort	1 (11.1)	0 (0.0)	1 (3.3)	4 (11.4)	0.050	3 (6.7)	0 (0.0)	1 (12.5)	1 (20.0)	1 (9.1)	0.399	1 (5.0)	2 (5.4)	3 (12.5)	0.158
Severe pain or discomfort	0 (0.0)	0 (0.0)	1 (3.3)	4 (11.4)		1 (2.2)	2 (16.7)	1 (12.5)	0 (0.0)	1 (9.1)		0 (0.0)	3 (8.1)	2 (8.3)	
Extreme pain or discomfort	0 (0.0)	0 (0.0)	0 (0.0)	0 (0.0)		0 (0.0)	0 (0.0)	0 (0.0)	0 (0.0)	0 (0.0)		0 (0.0)	0 (0.0)	0 (0.0)	
**Anxiety/Depression, n (%)**															
Not anxious or depressed	7 (77.8)	4 (57.1)	11 (36.7)	14 (40.0)		21 (46.7)	5 (41.7)	2 (25.0)	2 (40.0)	6 (54.6)		14 (70.0)	9 (24.3)	13 (54.2)	
Slightly anxious or depressed	0 (0.0)	1 (14.3)	16 (53.3)	13 (37.1)		18 (40.0)	4 (33.3)	4 (50.0)	2 (40.0)	2 (18.2)		4 (20.0)	22 (59.5)	4 (16.7)	
Moderately anxious or depressed	2 (22.2)	1 (14.3)	1 (3.3)	6 (17.1)	0.446	3 (6.7)	3 (25.0)	1 (12.5)	1 (20.0)	2 (18.2)	0.825	0 (0.0)	5 (13.5)	5 (20.8)	0.033
Severely anxious or depressed	0 (0.0)	1 (14.3)	1 (3.3)	1 (2.9)		2 (4.4)	0 (0.0)	1 (12.5)	0 (0.0)	0 (0.0)		1 (5.0)	1 (2.7)	1 (4.2)	
Extremely anxious or depressed	0 (0.0)	0 (0.0)	1 (3.3)	1 (2.9)		1 (2.2)	0 (0.0)	0 (0.0)	0 (0.0)	1 (9.1)		1 (5.0)	0 (0.0)	1 (4.2)	
